# How a fly photoreceptor samples light information in time

**DOI:** 10.1113/JP273645

**Published:** 2017-04-17

**Authors:** Mikko Juusola, Zhuoyi Song

**Affiliations:** ^1^ Department of Biomedical Science University of Sheffield Sheffield S10 T2N UK; ^2^ National Key laboratory of Cognitive Neuroscience and Learning Beijing Beijing Normal University Beijing 100875 China

**Keywords:** adaptive sampling, drosophila, information theory, photoreceptor, vision, quantum bump

## Abstract

A photoreceptor's information capture is constrained by the structure and function of its light‐sensitive parts. Specifically, in a fly photoreceptor, this limit is set by the number of its photon sampling units (microvilli), constituting its light sensor (the rhabdomere), and the speed and recoverability of their phototransduction reactions. In this review, using an insightful constructionist viewpoint of a fly photoreceptor being an ‘imperfect’ photon counting machine, we explain how these constraints give rise to adaptive quantal information sampling in time, which maximises information in responses to salient light changes while antialiasing visual signals. Interestingly, such sampling innately determines also why photoreceptors extract more information, and more economically, from naturalistic light contrast changes than Gaussian white‐noise stimuli, and we explicate why this is so. Our main message is that stochasticity in quantal information sampling is less noise and more processing, representing an ‘evolutionary adaptation’ to generate a reliable neural estimate of the variable world.

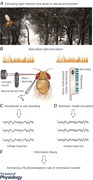

**Abbreviations**
GWN: Gaussian white‐noise
NS: naturalistic stimulation
R1–R6s: outer photoreceptors
R7 and R8: inner photoreceptors
TRP/TRPL: transient receptor potential/transient receptor potential like

## Introduction to adaptive quantal information sampling

**Figure 1:**
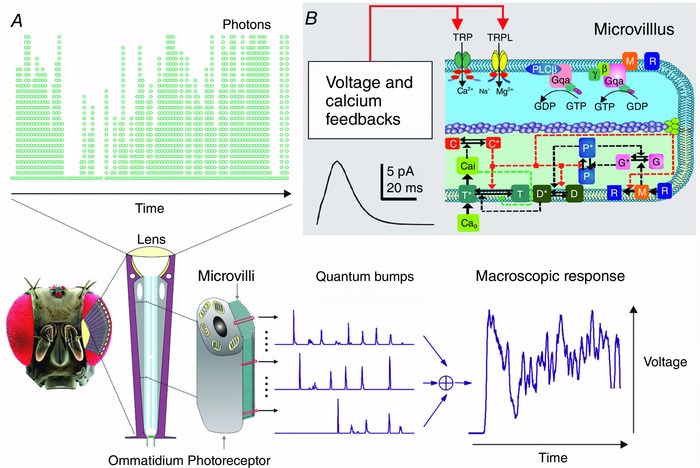
Schematic representation of adaptive quantal light information sampling by a *Drosophila* R1–R6 photoreceptor *A*, each R1–R6 samples photon influx by ∼30,000 microvilli, which together form its photosensitive light guide, the rhabdomere. Single‐photon responses (quantum bumps) from individual microvilli integrate a macroscopic response. *B*, top, each microvillus contains full phototransduction reactions, generating one quantum bump (sample) to an absorbed photon at a time; voltage and Ca^2+^‐dependent feedbacks regulate sample size and speed. Bottom, stochastic processes simulate bump generation. Molecular participants in microvillar phototransduction reactions: C, Ca^2+^‐dependent negative feedback to multiple targets; D, DAG; M, metarhodopsin; P, G protein‐PLC complex; T, TRP/TRPL channels (^*^, activated form). Red and green dotted arrows indicate negative and positive feedbacks, respectively, as used in the stochastically operating R1–R6 model (Song *et al*. 2012; Song & Juusola, 2014; Juusola *et al*. 2015). The gating mechanisms are yet unresolved, but these probably include production of DAG, Ins*P*_3_, proton, and physical microvilli contraction (Hardie & Franze, 2012).

A fly photoreceptor collects information about the world by counting photons within its receptive field (Fig. 1 *A*). These counts (samples) are its quantum bumps – small discrete transmembrane ion fluxes, produced by single microvilli (sampling units, Fig. 1 *B*) in response to single photons (Hardie & Juusola, 2015). A fruit fly (*Drosophila melanogaste*r) R1–R6 photoreceptor has ∼30,000 microvilli, each of which houses full phototransduction reactions. Collectively, the microvilli form the photoreceptor's light guide, the rhabdomere, and their quantum bumps, through stochastic size and timing variations (Fig. 1 *A*), integrate for each moment (time bin) its graded macroscopic response (output) to light intensity changes (input).

**Figure 2:**
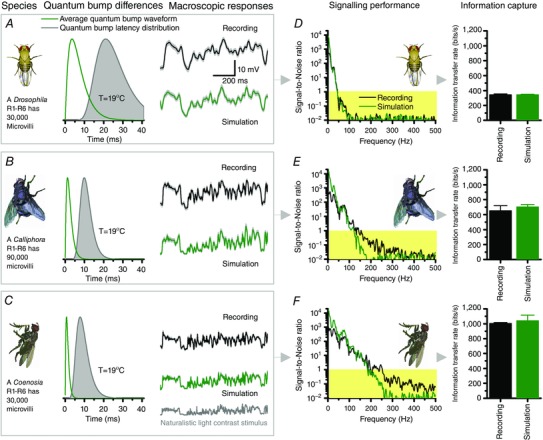
Photoreceptors that generate more, faster and more precise samples (quantum bumps) from the same light stimulus carry more visual information *A–C*, macroscopic voltage responses of fruit fly (*Drosophila*), blowfly (*Calliphora*) and killer fly (*Coenosia*) R1–R6 photoreceptors, respectively, to the same repeated naturalistic light intensity time series stimulus (NS) recorded *in vivo* and simulated by stochastic models. The number of microvilli (sampling units) and their average quantum bump waveforms (sample size; green) and latency distributions (sample jitter; grey) from *in vivo* recordings were used in the corresponding stochastic models, having no free parameters. The simulated voltage responses (green) to the NS behaved as their real counterparts (black). *D–F*, respective signal‐to‐noise ratios (SNR) and the corresponding information transfer rates of the simulated responses follow those of the real recordings. Data are from Song *et al*. (2012).

Based on information theory (Shannon, 1948), information in photoreceptor output depends upon the signal‐to‐noise ratio of its frequency‐domain representation (Fig. 2). This estimate, which measures the reproducibility of the underlying quantum bump size and rate changes (Juusola & Hardie, 2001a), can be inferred from high‐quality intracellular recordings to repeated light stimulation by signal and noise analyses (Juusola *et al*. 1994, 2016b; Juusola & Hardie, 2001a,*b*) and reproduced by stochastic simulations (Song *et al*. 2012; Song & Juusola, 2014, 2017; Juusola *et al*. 2015). Equally, this information is the difference between the output entropy and noise entropy rates (Shannon, 1948; Juusola & de Polavieja, 2003).

Experiments and simulations about light information sampling in R1–R6 photoreceptors of different fly species, which boast different microvilli numbers and quantum bump speeds (Fig. 2 *A–C*), have demonstrated that the photoreceptor's signalling performance (Fig. 2 *D–F*) increases as a function of quantum bump production rate. Specifically, the larger, the finer and the more precise a photoreceptor's bump rate changes are, the higher its information transfer rate. This means that fast flying *Calliphora* and *Coenosia* can extract more visual information from the same natural environment than a slow flying *Drosophila*, in which photoreceptors, respectively, have either fewer or slower microvilli and thus produce fewer, slower and more variable quantum bumps.

## Visual invariance emerging from adaptive quantal sampling

**Figure 3:**
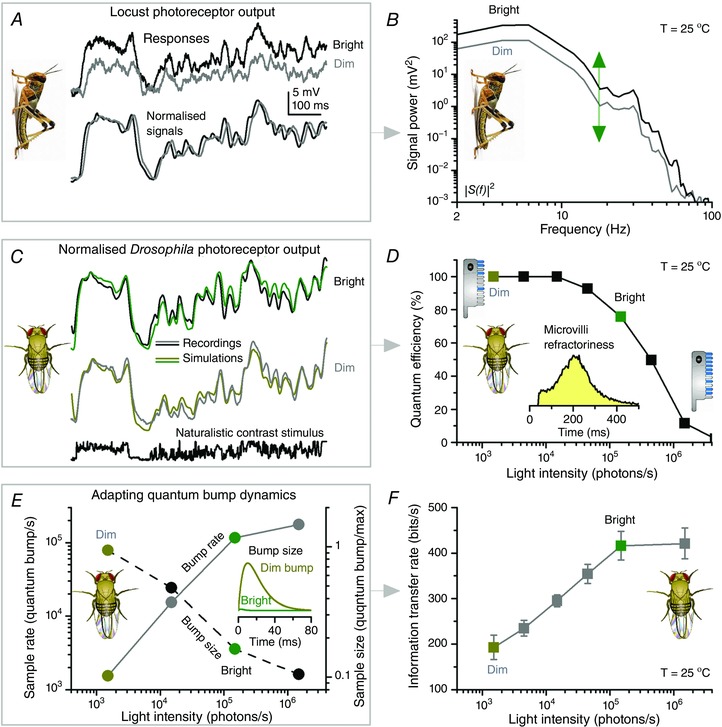
Quantal sampling adapts to provide invariable responses from natural contrast changes *A*, intracellularly recorded locust photoreceptor output to the same naturalistic contrast pattern has a similar waveform at dim (1,500 photons s^−1^; grey) and bright (1.5 × 10^5^ photons s^−1^; black) stimulation, implying that the same frequency range is utilised at different illumination. *B*, this is confirmed by the similar power spectra of the corresponding average responses, or signals (*n* = 100 repetitions). The arrow highlights the up‐shift in gain with brightening. *C*, normalised voltage signals (*n* = 100 repetitions) of both real and simulated *Drosophila* R1–R6 photoreceptors to the same naturalistic contrast pattern at dim and bright illuminations indicate comparable invariance. *D–F*, with brightening naturalistic stimulation: *D*, quantum efficiency (photon‐to‐bump conversion probability) decreases as more of a R1–R6 photoreceptor's 30,000 microvilli becomes refractory (insets), incapable of producing quantum bumps for the next 50–500 ms after their last photon hit. *E*, however, with more microvilli being activated, sample rate increases (continuous line) until progressive reduction in quantum efficiency *D*, stabilizes their quantum bump output. Simultaneously, sample size (bump waveform) is attenuated (dashed line). *F*, a photoreceptor's information transfer rate follows the increase in its quantum bump rate. Together, the adapting quantum bump dynamics ensure that relative changes in voltage responses represent naturalistic light changes (contrasts) accurately, irrespective of the ambient illumination. Although contrast gain in absolute terms (voltage/unit contrast) increases with light intensity, the temporal structure of the transmitted signal remains practically invariable. Data are from Faivre & Juusola (2008) and Song *et al*. (2012).

Nonetheless, the accurate behaviours of both diurnal and nocturnal insects (Esch *et al*. 2001; Gonzalez‐Bellido *et al*. 2011; Baird *et al*. 2015; Stürzl *et al*. 2016) suggest that insects perceive the world consistently, requiring their visual systems to generate highly invariable neural representations of natural images and objects over vastly (logarithmically) varying light conditions. Remarkably, this invariance is already clearly seen in both locust (Fig. 3 *A* and *B*) and fly photoreceptor (Fig. 3 *C*) outputs (Faivre & Juusola, 2008; Song *et al*. 2012; Friederich *et al*. 2016) and is mechanistically traceable to two central adaptations in quantal information sampling:

First, after a microvillus generates a quantum bump, it is briefly rendered refractory (full range: 50–500 ms; Fig. 3 *D*, inset), during which it cannot respond to another photon (Song *et al*. 2012). This progressively reduces quantum efficiency (Fig. 3 *D*; a photoreceptor's photon‐to‐bump‐conversion probability) with increasing light intensity, ultimately saturating sample (quantum bump) rate changes (Fig. 3 *E*, continuous line) to a given contrast stimulus (Song *et al*. 2012).

Second, during intense quantum bump production in bright illumination, the increased Ca^2+^ and Na^+^ influx through transient receptor potential (TRP)/transient receptor potential like (TRPL) channels reduces cationic driving force (Song *et al*. 2012) and the average bump size and duration (Figs 1 *B* and 3 *E*, inset and dotted line) (Henderson *et al*. 2000; Juusola & Hardie, 2001a). Photoreceptor output is further smoothened by quantum bump jitter (latency distribution; Fig. 2 *A*, grey), caused by stochasticity in the microvillar phototransduction reactions (Henderson *et al*. 2000; Juusola & Hardie, 2001a; Song *et al*. 2012).

Consequently, with the average quantum count (Fig. 3 *E*, continuous line) and bump size (dotted line) adapting to mean light intensity, the resulting macroscopic photoreceptor voltage response to a given natural contrast stimulus scales to look similar at different illumination conditions (Faivre & Juusola, 2008; Song *et al*. 2012; Friederich *et al*. 2016) (Fig. 3 *A–C*). These adapting quantum bump dynamics thus much contribute to the divisive nonlinearity, associated with amplitude normalisation in empirical photoreceptor models (French *et al*. 1993; van Hateren & Snippe, 2006; Friederich *et al*. 2016), providing mechanistic insight into descriptive nonlinear systems identification. [*Note: Divisive nonlinearity is an arbitrary but necessary mathematical operation in empirical (black‐box) photoreceptor models to compress vast light input changes into their limited output range. It makes the model output better approximate the real photoreceptor output*.] But while the average light intensity increasing over a range of magnitudes progressively reduces the photoreceptor's quantum efficiency (Fig. 3 *D*), the macroscopic responses still count in more quantum bumps (samples) (Fig. 3 *E*). This increase in their sample rate changes (from the same contrast) is the main reason why an insect photoreceptor's signal‐to‐noise ratio and information transfer (Fig. 3 *F*) increases with brightening until saturation (Juusola *et al*. 1994; Juusola & Hardie, 2001a; Faivre & Juusola, 2008; Frederiksen *et al*. 2008; Heimonen *et al*. 2012; Song & Juusola, 2014), when about half of its microvilli become refractory (Song *et al*. 2012). [*Note: in dim stimulation, a photoreceptor may count 10 quantum bumps (samples) in time‐bin_1_ and 2 in time‐bin_2_. But in brighter stimulation, time‐bin_1_ may have 100 and time‐bin_2_ 20 samples. Thus, the corresponding sample rate changes would be 8 and 80 samples/time, with the brightening increasing their difference by 10‐fold. The larger the sample rate changes, the higher the entropy rate. And if reproducible (having low noise), then the larger sample rate changes have a higher signal‐to‐noise ratio and transmit more information*.]

Thus, at the level of sampling, the neural code of insect photoreceptors inherently emphasises natural contrast constancy (relative light changes in the environment remain the same in different illumination conditions) (Attneave, 1954; Barlow, 1961; van Hateren, 1997) and efficiently allocates this information as invariable response waveforms within their limited output range (Atick, 1992; van Hateren, 1992b). And it does this, unavoidably, at the expense of coding the absolute light intensity.

Markedly, however, losing photons galore to refractory microvilli, when the flux of incident photons into distinct receptive fields in daylight can be 10^6^–10^9^ photons s^−1^, is not critical for good vision. As long as a *Drosophila* R1–R6 photoreceptor counts up ∼15,000–150,000 quantum bumps s^−1^, its neural estimate of local contrast changes will be reliable (of very high signal‐to‐noise ratio), with each photoreceptor in the eye providing hundreds of bits of information per second to the brain.

Functional comparisons in light information processing and representation between different insect photoreceptors suggest that perhaps all rhabdomeric photoreceptors would sample quantal light information similarly. Yet, by evolving different microvilli numbers and phototransduction speeds (Fig. 2), photoreceptors of different species have specialised visual capabilities for different life‐styles and habitats (Wong *et al*. 1982; van Hateren, 1992a; Juusola *et al*. 1994; van Steveninck & Laughlin, 1996; Anderson & Laughlin, 2000; Juusola & Hardie, 2001a; Niven *et al*. 2007; Faivre & Juusola, 2008; Frederiksen *et al*. 2008; Gonzalez‐Bellido *et al*. 2011; Frolov *et al*. 2012; Heimonen *et al*. 2012; Song *et al*. 2012; Song & Juusola, 2014; Song *et al*. 2016). Here, the trade‐off is that while having more microvilli increases photoreceptor output, its bandwidth, and information transfer rate for representing natural contrast changes (Song & Juusola, 2014), so does the total cost for constructing, maintaining and running this sampling machinery (Laughlin *et al*. 1998; Song & Juusola, 2014).

Future work is needed to test how these sampling rules and constraints apply to nocturnal moths, which have high microvillus numbers but seemingly noisy macroscopic responses (Stockl *et al*. 2016) and possibly inferior information transfer rates compared to diurnal insects with fewer microvilli. Moreover, an open question remains of how the refractory light information sampling dynamics of microvillar photoreceptors compare mechanistically with bleaching adaptation of vertebrate ciliary photoreceptors (Yau & Hardie, 2009).

## Antialiasing through quantal adaptive sampling

Interestingly, stochastic quantal information sampling is not only an elegant light‐adaptation strategy but possibly also an evolutionary solution to the temporal aliasing problem to provide reliable neural estimates of the variable world (Juusola *et al*. 2015). It scatters high‐frequency information into broadband noise rather than generating the false patterns produced by regular sampling (Dippe & Wold, 1985). Thus, variable sampling times and sample sizes (quantum bump jitter and size differences) prevent distortions or artefacts, such as harmonic oscillations (Song *et al*. 2012), in reconstruction of macroscopic responses from the original (continuous) light patterns. And because the flies have neural superposition eyes, which provide eight independent estimates (outer R1–R6 and inner R7 and R8 photoreceptors) of local light intensity changes for each image pixel (Kirschfeld & Franceschini, 1969; Horridge & Meinertzhagen, 1970), parallel sampling by microvilli in each photoreceptor and the later synaptic pooling of their macroscopic outputs would actively cancel noise.

In support of this view, stochastic modelling implies that intrinsic noise – caused by the quantum bump variations – seems to degrade the fly photoreceptor output less than what was thought before (Lillywhite & Laughlin, 1979; Laughlin & Lillywhite, 1982), maximally ∼5–10% (Song *et al*. 2012; Song & Juusola, 2014; Juusola *et al*. 2015). The concerted action of many thousands of microvilli in photon sampling reduces intrinsic noise as their quantum bumps add up the macroscopic response. Moreover, global (intracellular calcium and membrane voltage) feedbacks (Fig. 1 *B*), which carry memory of the past events, reduce noise by adapting the bump sizes to the ongoing light stimulation. This accounts for ∼10% improvement in the rate of information transfer, in comparison to sampling the bumps randomly from the same distribution (Song *et al*. 2012). Intrinsic noise is almost certainly further reduced when the parallel macroscopic responses of photoreceptors, which view the same point in space, are pooled in convergent synaptic transmission to interneurons (Zheng *et al*. 2006).

Here, the key realisation is that integration of variable samples, which are much briefer than the world structure they encode in time, increases information and reduces noise, improving the robustness, reliability and accuracy of the resulting neural estimates (see also: Galton, 1907; Heimonen *et al*. 2006; Padmanabhan & Urban, 2010). Conversely, filtering responses downstream, which occurs by a voltage‐sensitive cell membrane (Hardie, 1991; Weckstrom *et al*. 1991; Vähäsöyrinki *et al*. 2006), can reshape and smoothen photoreceptor output but not increase its information (data processing theorem) (Shannon, 1948; Juusola & de Polavieja, 2003; Abou Tayoun *et al*. 2011).

Armed with this essential mechanistic knowledge about fly photoreceptors’ stochastic refractory photon sampling, we next briefly consider why and how this makes encoding inefficient for Gaussian white‐noise (GWN) stimulation but sensitised to salient natural world features.

## Gaussian white‐noise stimulation does not fully test photoreceptor performance

In electronic systems, GWN stimulus, which maximises information within its bandwidth and variance, is regularly used for testing information transmission capacity. However, for neural systems, such as photoreceptors, which employ adaptive quantal information sampling, GWN fails to test their true signalling performance (Rieke *et al*. 1995; Juusola & de Polavieja, 2003; Song & Juusola, 2014).

**Figure 4:**
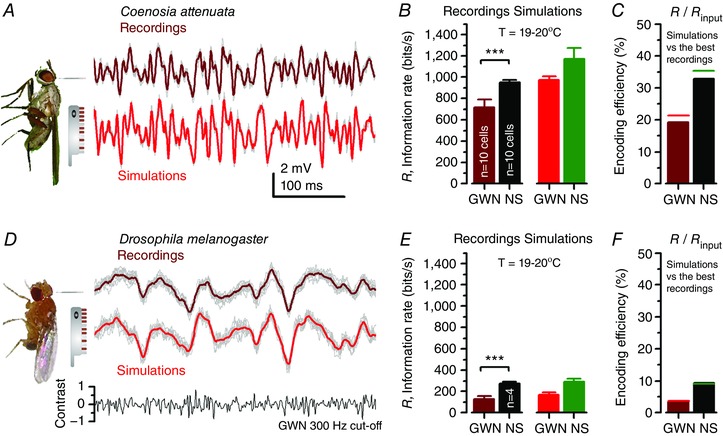
Fly photoreceptors encode Gaussian white‐noise (GWN) and naturalistic stimuli (NS) differently, with different efficiencies and costs Both fast‐flying *Coenosia* and slow‐flying *Drosophila* photoreceptors have 30,000 microvilli, but those of *Coenosia* sample light changes and recover from them faster, resulting in higher information capture. *A*, voltage responses of a *Coenosia* R1–R6 photoreceptor (brown) and respective stochastic model simulations (red) to unit‐contrast GWN stimulation with 300 Hz cut‐off; light level: ∼10^6^ photons s^−1^. *B*, information transfer of recorded and simulated *Coenosia* R1–R6 voltage responses to GWN stimulus and naturalistic stimulation (NS; see Fig. 2); these cells capture ∼20% less information from the GWN than NS. *C*, overall, *Coenosia* R1–R6s encoded ∼30% of information in NS; performing ∼1.7 times more efficiently than with 300 Hz GWN. *D*, *Drosophila* R1–R6 voltage output (brown) and respective stochastic model simulations (red) to the same unit‐contrast GWN stimulation as in *A*. *E*, information transfer rates of recorded and simulated *Drosophila* R1–R6 voltage responses to GWN (*D*) and NS; these cells capture ∼60% less information from the GWN than NS. Moreover, *Drosophila* R1–R6s encode both the stimuli less efficiently than *Coenosia* R1–R6s. *F*, *Drosophila* photoreceptors encoded NS ∼2.5 times more efficiently than 300 Hz GWN. In every cell, NS evoked higher information transfer. Here simulated *Coenosia* photoreceptor output carries proportionally more information than the average recordings because it is based on the best GWN and NS recordings. Simulations lack recording noise and muscle activity, which reduce information in recordings, and the intracellular pupil mechanism. Data are from Song & Juusola (2014).

This disparity has less to do with the used GWN's absolute energy, phase distribution or photon content, but primarily depends upon how a photoreceptor samples photons (Song & Juusola, 2014). Therefore, a bright GWN stimulus with a mean intensity that is 100‐fold higher than the mean intensity of a naturalistic stimulus would still yield lower information transfer estimates in a photoreceptor. This is because stochastic photon sampling by finite refractory microvilli populations makes a photoreceptor to encode different stimulus statistics differently, with different efficiencies and costs (Fig. 4). Specifically, longer dark contrasts, which characterize naturalistic stimuli, help to recover more refractory microvilli than equally bright stimuli without these features, improving neural information while lowering its metabolic costs (Song & Juusola, 2014). Photoreceptor output to natural/naturalistic stimulation thus typically entails larger phasic quantum bump rate changes, while maintaining a lower mean level of depolarization (costing less) than corresponding responses to GWN (Juusola & de Polavieja, 2003; Song & Juusola, 2014). Conversely, GWN experiments underestimate a photoreceptor's information transmission capacity, while often overestimating its normal energy consumption (Song & Juusola, 2014), making the subsequent neuro‐economics estimates and their cross‐species comparisons unrealistic.

For example, at 25°C, a typical *Drosophila* R1–R6 photoreceptor would sample 282 bits s^−1^ from 200 Hz band‐limited GWN, costing 1.31 × 10^7^ ATP molecules bit^−1^. However, from a rich naturalistic contrast stimulus of equal mean brightness, the same photoreceptor would sample 455 bits s^−1^ with the price of 1.15 × 10^7^ ATP molecules bit^−1^ (Song & Juusola, 2014).

## Adaptive quantal sampling enhances salient stimuli

Importantly, adaptive quantal sampling gives a fly photoreceptor an innate capacity to enhance stimulus salience. Adaptation that follows the phototransduction cannot increase the photoreceptor's information transfer rate (Juusola & de Polavieja, 2003). However, if the receptor adapts during the process of sampling, it can accentuate quantum bump rate changes to new (surprising) stimuli, increasing information transfer rate transiently.

**Figure 5:**
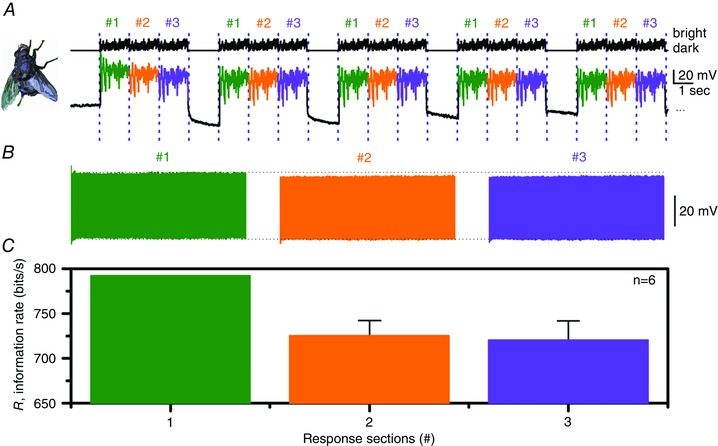
Quantal adaptive sampling innately accentuates salient contrast changes, boosting their information content *A*, upper traces, a bright light stimulus consisting of 3 identical naturalistic intensity sequences, each lasting 1 s and numbered 1, 2 and 3, followed by a 1 s‐long dark period is repeated 1000 times. Lower traces, a typical *Calliphora* R1–R6 photoreceptor voltage response to this stimulus. *B*, the photoreceptor responses for these three groups are separated and grouped retaining the timing order. Notice that the responses to the first naturalistic stimulus sequence are slightly larger than the responses to the second and third stimulus sequences. *C*, the average information transfer rate of the responses during the three stimulus sequences. Voltage responses to the 1st stimulus sequence carry more information than those to the 2nd and 3rd sequences. This behaviour was consistent in all the recordings (*n* = 6) giving the first second of responses on average 9.5% higher information transfer rates. Data are from Juusola & de Polavieja (2003).

Naturalistic stimulation includes longer dark contrasts that are not present in GWN stimulation. The effect of short‐term adaptation to these events was first quantified by showing how *Calliphora* R1–R6s’ information transfer changes during three identical naturalistic stimulus sequences (Fig. 5 *A*; marked 1, 2 and 3) that followed a 1 s dark period (Juusola & de Polavieja, 2003). In repetitive stimulation, the responses to the 1st sequence (green) were always larger (Fig. 5 *B*) and carried more information (Fig. 5 *C*) than the responses to the 2nd and 3rd sequences. Thus, the signalling precision of fly photoreceptors was higher at transitions from dark to bright light and then reduced with adaptation to a lower voltage response.

Further recordings and photoreceptor model simulations have since demonstrated that the first larger response to a bright step simply contains more quantum bumps, and thus has a higher signal‐to‐noise ratio than subsequent responses, for which fewer microvilli are activated, with more microvilli becoming refractory (Juusola & de Polavieja, 2003; Song *et al*. 2012; Song & Juusola, 2014). Similarly, the first negative voltage response to a dark contrast step will be larger (Juusola, 1993) because more microvilli will be refractory, generating fewer quantum bumps than the subsequent responses. Accordingly, the photoreceptors’ information transfer is higher at large dark‐to‐bright or bright‐to‐dark contrast transitions and decreases afterwards in correlation with the adaptation to the stimulus (Juusola & de Polavieja, 2003; Zheng *et al*. 2006, 2009).

Thus, not only does adaptive quantal sampling lead to robust encoding of natural light changes over the full dynamic range of environmental light intensities (Fig. 3) (Faivre & Juusola, 2008; Song *et al*. 2012, 2016; Friederich *et al*. 2016; Juusola *et al*. 2016a), it also enhances novel or surprising stimuli, which generate the largest quantum bump rate changes (increments or decrements) with respect to the ongoing average (Juusola & de Polavieja, 2003; Song *et al*. 2012; Song & Juusola, 2014) (Fig. 5). Remarkably, further analyses have shown that while accentuating saliency, adaptive quantal photon sampling also improves the allocation of information in naturalistic stimulation on the photoreceptors’ limited bandwidth and amplitude range (van Hateren, 1997; Zheng *et al*. 2009; Song *et al*. 2012; Juusola *et al*. 2016a). As the output frequency distribution flattens (or whitens) while its amplitude distribution becomes Gaussian, every symbol (voltage value) of a message (macroscopic voltage response) would be transmitted equally often (Shannon, 1948).

## Discussion

In this review, we have presented a basic account of how a fly photoreceptor samples light information in time, and why this improves vision. For clarity, the focus was upon stochastic adaptive photon sampling to highlight its fundamental role in generating reliable macroscopic responses to environmental light contrast changes. This meant that the primary sampling process was considered in isolation.

While the description given is accurate, the reality is more complex and in perpetual motion, and we know that optimal visual information sampling, at least in *Drosophila*, further involves elaborate photomechanical adaptations and self‐motion (body, head and eye movements), which prevent retinal images from fading during fast adaptation (Juusola *et al*. 2016a). In fact, during photon sampling, light input intensity is regulated by two photomechanical processes inside photoreceptors. Slower screening pigment migration (intracellular pupil, 1–10 s) (Franceschini & Kirschfeld, 1971, 1976) and much faster autonomous microsaccadic photoreceptor contractions (0.01–1 s) (Hardie & Franze, 2012; Juusola *et al*. 2016a) dynamically reduce photon flux into the rhabdomere, shifting and narrowing its receptive field (Juusola *et al*. 2016a). In addition, downstream, in the photoreceptor axons, asymmetric synaptic and gap‐junctional inputs from the network differentiate individual R1–R6 outputs (Shaw, 1984; Shaw *et al*. 1989; Zheng *et al*. 2006, 2009; Nikolaev *et al*. 2009; Rivera‐Alba *et al*. 2011; Wardill *et al*. 2012; Dau *et al*. 2016).

How all these factors contribute to spatiotemporal encoding of the visual world and perception, providing *Drosophila* hyperacute vision, is analysed in detail in (Juusola *et al*. 2016a) and is beyond the scope of this review.

## Additional information

### Competing interests

The authors declare no conflict of interest.

### Author contributions

M.J. and Z.S. wrote the paper. M.J. and Z.S. approved the final version of the manuscript and agree to be accountable for all aspects of the work. All persons designated as authors qualify for authorship, and all those who qualify for authorship are listed.

### Funding

This work was supported by the open research fund of the State Key Laboratory of Cognitive Neuroscience and Learning (M.J.), NSFC project (30810103906: M.J.), Jane and Aatos Erkko Foundation (M.J.), The Leverhulme Trust grant (RPG‐2012‐567: M.J.) and the BBSRC grants (BB/F012071/1, BB/D001900/1 and BB/H013849/1: M.J.). EPSRC‐funded 2020 Science fellowship (EP/I017909/1: S.Z.) provided the major funding for the ‘1st UK Phototransduction and Synaptic Transmission’ workshop.
